# When Intimate Partner Violence Meets Same Sex Couples: A Review of Same Sex Intimate Partner Violence

**DOI:** 10.3389/fpsyg.2018.01506

**Published:** 2018-08-21

**Authors:** Luca Rollè, Giulia Giardina, Angela M. Caldarera, Eva Gerino, Piera Brustia

**Affiliations:** Department of Psychology, University of Torino, Turin, Italy

**Keywords:** same sex intimate partner violence, same-sex couple, LGB, domestic violence, IPV, treatment

## Abstract

Over the past few decades, the causes of and intervention for intimate partner violence (IPV) have been approached and studied. This paper presents a narrative review on IPV occurring in same sex couples, that is, same sex IPV (SSIPV). Despite the myth that IPV is exclusively an issue in heterosexual relationships, many studies have revealed the existence of IPV among lesbian and gay couples, and its incidence is comparable to (Turell, 2000) or higher than that among heterosexual couples (Messinger, 2011; Kelley et al., 2012). While similarities between heterosexual and lesbian, gay, and bisexual (LGB) IPV were found, unique features and dynamics were present in LGB IPV. Such features are mainly related to identification and treatment of SSIPV in the community and to the need of taking into consideration the role of sexual minority stressors. Our findings show there is a lack of studies that address LGB individuals involved in IPV; this is mostly due to the silence that has historically existed around violence in the LGB community, a silence built on fears and myths that have obstructed a public discussion on the phenomenon. We identified the main themes discussed in the published studies that we have reviewed here. The reviews lead us to the conclusion that it is essential to create a place where this subject can be freely discussed and approached, both by LGB and heterosexual people.

## Introduction

Over the past few decades, intimate partner violence (IPV) has received increasing interest from mental health experts. According to the World Health Organization (2012), IPV is related to any behavior between a couple that involves acts of physical and sexual violence, emotional and psychological abuse, and controlling behavior. According to numerous authors, the expression “IPV” represents a form of violence that both men and women can enact, with no regard to age, marital status, or sexual orientations (Capaldi et al., 2007; Ali et al., 2016). The consequences of IPV on mental health and general wellbeing have also been outlined in numerous studies (Campbell, 2002; Anderson et al., 2008; Murray and Mobley, 2009; Giordano et al., 2014; Costa et al., 2015).

The lesbian, gay, and bisexual (LGB) population faces more difficult outcomes compared to the heterosexual population “across different life domains, including mental and physical health, subjective wellbeing, employment, poverty, homelessness, and social exclusion” (Perales and Todd, 2018, p. 190). IPV in the LGB population has not been studied as frequently as in the heterosexual population: in 2015, research on LGB IPV constituted a mere 3% of the total research on the subject (Edwards et al., 2015). Even though there are a few studies on Same-Sex Intimate Partner Violence (SSIPV), they highlight that the phenomenon occurs at a rate that is comparable (Turell, 2000) or even higher than heterosexual IPV (Messinger, 2011; Kelley et al., 2012; Barrett and St.Pierre, 2013). It can be difficult to identify LGB IPV prevalence rates due to the different methodologies used in the researches. However, according to one of the most recent and representative study reports, almost one-third of sexual minority males and one-half of sexual minority women in the United States affirmed they were victims of physical or psychological abuse in a romantic relationship. In addition, around 60% of gay men and 63% of lesbian women reported that they experienced psychological IPV (Breiding et al., 2013). Breiding et al. (2013) identified that 4.1 million people of the LGB community have experienced IPV in their lifetime in the United States.

Life-time prevalence of IPV in LGB couples appeared to be similar to or higher than in heterosexual ones: 61.1% of bisexual women, 43.8% of lesbian women, 37.3% of bisexual men, and 26.0% of homosexual men experienced IPV during their life, while 5.0% of heterosexual women and 29.0% of heterosexual men experienced IPV. When episodes of severe violence were considered, prevalence was similar or higher for LGB adults (bisexual women: 49.3%; lesbian women: 29.4%; homosexual men: 16.4%) compared to heterosexual adults (heterosexual women: 23.6%; heterosexual men: 13.9%) (Breiding et al., 2013).

Messinger (2011) highlighted that all forms of abuse were more likely to occur in homosexual and bisexual couples than in heterosexual ones. Moreover, he hypothesized that a higher percentage violence was caused by unique risk factors linked to minority stress that is experienced only by LGB people. In addition, the study highlighted that lesbian women were at higher risk of being involved in IPV, followed by heterosexual women, gay men, and heterosexual men. Furthermore, bisexual people appeared to be the most abused group compared to the others; bisexual women, specifically, were more likely to be victims of every type of IPV, excluding psychological IPV.

Most researches on the prevalence of SSIPV have been conducted on a North American population, while some minor studies are focused on Australian (Leonard et al., 2008), Chinese (Chong et al., 2010; Liu et al., 2013), South African (Eaton et al., 2013), and British populations (Guasp, 2012): the results reported similar or even higher IPV rates compared to those for North American populations. Chard et al. (2012), in their transnational research, evidenced the differences in prevalence rates among various countries: participants were recruited through advertisements on Facebook in the United States, Canada, Australia, United Kingdom, Republic of South Africa (RSA), Brazil, Nigeria, Kenya, and India. Their findings showed similar rates between United States and the other nations, while the rate of physical abuse appeared to be similar or more likely to occur in Australia, Brazil, Republic of South Africa, and the United Kingdom than in the United States.

In Italy, two studies were conducted on lesbian IPV—one by Moscati (2016) (as part of a European project) and a survey by Arcilesbica (2011). Moscati (2016) work was mainly focused on the absence of protective laws for lesbian women victims of IPV, and Arcilesbica (2011) attempted to estimate IPV prevalence among Italian lesbian women. The sample comprised 102 lesbian women, mostly Italian (88.2%). Participants answered a questionnaire containing 29 multiple-choice questions. In over one case out of five (20.6% of the total), the interviewee admitted to be afraid of her partner coming back home. Further, 41.2% of women occasionally hid something from their partners because they were afraid of their reactions. In addition, 14.7% of lesbian women declared that they were always afraid of their partners. Almost half of the interviewees identified the damage resulting from a couple fight as psychological; physical damage was reported by 5.9% of the interviewees (Arcilesbica, 2011).

In the light of such findings, it is apparent that LGB IPV needs to be studies further. Nonetheless, public opinion considers LGB abuse as a rare phenomenon: this opinion is particularly strong with regard to bisexual and lesbian women, often idealized as being in peaceful and utopian relationships, far from the violence and aggression that is commonly associated with “typical” male virility (Glass and Hassouneh, 2008; Barnes, 2010). Such a stereotype can be an obstacle to lesbian victims in recognizing that a partner behavior is abusive and not normal (Seelau and Seelau, 2005).

Previous research has suggested the need of further research on the issue: LGB IPV has a double invisible nature that is responsible of the lack of studies on it. In the past, health experts found many obstacles in accessing research and data on SSIPV, a fact that implicated negative consequences in terms of prejudice and misinformation in addition to the more obvious outcomes (Messinger, 2011).

## Aims

In the light of the background outlined above, this paper presents a narrative review aimed at (1) providing an overview, through a selective narrative review, of the psychological literature on LGB IPV, with a specific focus on treatments and interventions addressed both to victims and perpetrators, and (2) identifying, from the literature, suggestions for future directions in research for LGB-oriented psychological and community services in relation to IPV and the themes outlined by the overview.

## Method

A literature research was conducted by using the following databases: PsycINFO, PsycARTICLES, PubMed, and Google Scholar. The search criteria was that eligible studies should have been published in English or Italian, between 1995 and 2017, and focused on the main features of LGB IPV.

The following combinations of keywords were used to conduct the research: (1) Same-sex intimate partner violence OR, SSIPV OR, LGB intimate partner violence OR, LGB IPV; (2) Same-sex domestic violence OR, LGB domestic violence; (3) Lesbian domestic violence; (4) Gay domestic violence; (5) Bisexual domestic violence; (6) Prevalence; (7) Minority stress; (8) Treatment; and (9) Intervention.

**Table 1** presents the selection criteria applied to select the papers.

**Table 1:** Inclusion and exclusion criteria.

Inclusion criteria	Exclusion criteria
(a) Researches published between 1995 and 2018	(a) Researches published before 1995
(b) Researches published in English or Italian languages	(b) Researches published in languages other than English and Italian
(c) Focus on SSIPV	(c) Contributions from books or sources different from published articles
(d) Include combinations of search terms and key words listed in the Method section	and surveys.

We created a dataset of the selected papers and conducted a thematic analysis (TA) in order to outline patterns of meaning across the reviewed studies (Braun and Clarke, 2006), using a semantic approach. Braun and Clarke (2006) provided guidelines for conducting the TA, which included a process organized in six phases: (1) *Familiarization with the data*; (2) *coding*; (3) *searching for themes*; (4) *reviewing themes*; (5) *defining and naming themes*; and (6) *writing up*.

Thus, after the *Familiarization* phase, we assigned each article with a short label that identified the main results that could be relevant to our aims (*Coding* phase). Thereafter, we identified broader patterns of meaning, each representing a candidate theme to which the papers were allocated. Next, we stepped into the *Reviewing themes* phase and checked back the candidate themes confronting them with the studies dataset. We attempted to define more inclusive thematic areas by combining specific candidate themes and by selecting a pool of the most frequent ones, which led us to the *Defining and naming themes* phase. As a result of this process, we identified the six main themes that were focused on in the studies: *silence around the phenomenon*; *association with Sexual Minority Stress*; *assessment and treatment*; *couple and group interventions*; *victims’ treatmen*ts; and *access to services offering help and support*.

## Results

The first outcome of the research included 4700 sources, from which we eliminated duplicates, researches published in languages other than English and Italian, contributions from books or sources other than published articles and surveys. After this selection process, 119 studies met the inclusion criteria for this review.

### Silence Around Violence

Understanding LGB IPV prevalence and related factors may be difficult because of the silence that has historically existed around violence in the LGB community. Research has revealed that in the LGB community, several common fears became an obstacle for a public discussion on the phenomenon. For example, an aspect frequently claimed was that recognizing IPV in the LGB community may be used to stigmatize the community itself, thereby contributing to building additional oppression and social marginalization (Kaschak, 2001; Ristock, 2003). Similarly, the feminist community was averse to discussing the phenomenon, particularly when it involved lesbian couples: a public discussion on lesbian IPV may increase negative reactions to feminism and female homosexuality; on the other hand, it may minimize the concern regarding male violence against women (McLaughlin and Rozee, 2001; Ristock, 2001, 2003).

Furthermore, culturally created ideologies regarding masculinity and femininity may discourage IPV victims from openly discussing their experience. This happens when the perceived stigma reinforces their own stereotype that homosexual men are less masculine than heterosexual men, or the one that lesbian IPV is harmless (because women are not physically strong and dangerous) (Ristock and Timbang, 2005). Buttell and Cannon (2015) stated that IPV was not about genders, but more about power and control dynamics; thus, to assess and treat IPV, particularly LGB IPV, it is pointless to take into account gender-related stereotypes (Brown, 2008; Little and Terrance, 2010). However, the main resistance from the feminist community came from the risk that discussing lesbian IPV may threaten a feminist belief regarding women’s abuse, usually perpetrated by men who are influenced by misogyny and patriarchy. Gender and power were the main factors in this theory; therefore, lesbian victimization was considered both impossible (because of the inconsistency due to the absence of a man in the equation) or explained by the assimilation among lesbian women of misogyny and homophobia, which is subsequently projected on to their partners as women and homosexuals (Ristock and Timbang, 2005).

Many LGB individuals experienced additional victimization and homophobia when they reported the abuse to police (Barnes, 1998; Burke et al., 2002; Bentley et al., 2007; Guadalupe-Diaz and Yglesias, 2013). The LGB community Legal Rights and Protection Laws are intended to protect the LGB community (Moscati, 2016).

Bunker Rohrbaug (2006) indicated that one of the most pervasive and alarming myth was considering violence as a mutual conflict, particularly when the violence occurred in a gay couple, because men “fight equally,” as they are assumed to have comparable physical strength. This myth was legitimized by the societal attitude with regard to tolerating violence expressions between men, expressions that were considered admissible and often normalized as a means of dispute resolution or because of greater congruence between violence and male roles (Baker et al., 2013).

This idea implicated serious issues because not only did it created obstacles in providing services for homosexual victims but it also contributed to increasing the tendency to minimize IPV severity (McClennen, 2005). Such an assumption could neglect the study of other types of violence apart from the physical one, such as psychological abuse (Finneran and Stephenson, 2013). One of the reasons for why the “mutual fight myth” was so pervasive is related to researches that reported how common it was for a partner to be violent (Carvalho et al., 2011; Edwards and Sylaska, 2013). This myth was proved to be unfounded when motivations why partners fight back were considered. In this regard, several researches (Merrill and Wolfe, 2000; Bartholomew et al., 2006) attested that self-defense was the most common cause that victims reported to justify their fighting back. Further studies (Bartholomew et al., 2008; Little and Terrance, 2010; Bimbi et al., 2011) also investigated the victim fighting-back phenomenon and suggested that, because of the mutuality, the distinction between survivor and perpetrator might be less clear in LGB communities. Ristock (2001) affirms that fighting back was not only self-defense but also a claim to power and higher position between the couple. A further hypothesis supposed that additional and hidden power dynamics may contribute to the occurrence of IPV. These issues reinforced the illusion that violence was mutual (Ristock and Timbang, 2005). Moreover, the belief that it would be easier for gay men to leave an abusive relationship needs to be considered. This idea arose from another stereotype related to homosexual men being unable to be involved in a stable relationship and often and easily changing partners instead LGB relationship can be as stable as heterosexual ones (Gates, 2015).

Several studies (Austin et al., 2002; Girshick, 2002; Balsam and Szymanski, 2005; Bornstein et al., 2006; Messinger, 2011; Galletly et al., 2012) claimed how bisexual people experienced an additional stress related to IPV because of the lack of support from the LGB community. Bisexual people were doubly marginalized, not being recognized by lesbian and gay people as part of their community and, simultaneously, being stigmatized by heterosexuals. The assumption that bisexual people use the heterosexual privilege leads to the fact that a lot of lesbian and gay people believe that the victimization of bisexual people is not as serious as that of lesbian and gay people. Davidson and Duke (2009) showed that bisexual people were victims of the law system and the services to the same extent. Moreover, studies showed that biphobia within the LGB community increased the risk of IPV between bisexual partners and, simultaneously, reduced help-giving resources (Austin et al., 2002; Girshick, 2002; Balsam and Szymanski, 2005; Bornstein et al., 2006; Messinger, 2011; Galletly et al., 2012).

Without overlooking the peculiar aspects of the LGB community, authors compared the general patterns, types, impact and cycle of violence of LGB IPV and heterosexual IPV (McLaughlin and Rozee, 2001; Hequembourg et al., 2008). Like heterosexual victims, homosexual and bisexual people experienced emotional, physical, and sexual abuse. The outcomes were severe, and included physical injury, social isolation, property destruction and loss, and disruption to work, education, and career development (Buford et al., 2007; Chard et al., 2012; Barrett, 2015). Moreover, victims often reported that the abuse was not mutual and was instead suffered, and the consequences of it made them feel trapped, hopeless, and isolated (Ferraro and Johnson, 2000; McClennen, 2005). There were also similarities with regard to the reasons for remaining with the abusive partner. Both heterosexual and homosexual victims commonly listed the following aspects as reasons to stay: love for the partner, financial and emotional dependency on the partner, (Merrill and Wolfe, 2000). A further resemblance was the connection between stress, violence, and use of substances (Buford et al., 2007; Cain et al., 2008): IPV was related both to depression and substance use among LG people with a previous IPV history, who appeared to have a higher tendency of drug abuse (Kelley et al., 2011).

Gill et al. (2013) highlighted that the higher prevalence rate of HIV in the LGB population also constituted an important difference in their experience of IPV. Merrill and Wolfe (2000) results showed that the main reasons why HIV-positive IPV victims did not leave the relationship were linked to the fear of becoming sick and dying alone or of dating in the context of the disease. On the other hand, HIV-positive partners remained in the relationship because they did not want to abandon their sick partners. The authors argued that IPV increased vulnerability to risks associated with HIV transmission, which in turn affects medical care, mental health, adherence to therapy, frequency of follow-up; in addition, they found that IPV contributed per se to HIV transmission, directly through forced unprotected sex with a partner or indirectly by impairing the victim’s ability to negotiate safer sex. Individuals may experience difficulties in negotiating safer sex for several reasons, including the perception of being unable to have control over sex, fear of violence, and unequal power distributions in the relationship (Bowen and Nowinsky, 2012; Gill et al., 2013). In light of these data, it can be said that IPV may be common among people living with HIV. Therefore, it is essential that all service providers screen and provide assistance for issues relating to safer sex, similarly, all HIV service providers should screen for IPV and discuss safety within the context of abusive relationships and helping their clients have safer sex (Heintz and Melendez, 2006).

Even though this fact represented an issue in the heterosexual population, LGB people were more affected by it. In fact, in Merrill and Wolfe (2000) study the lack of knowledge about IPV was the third most commonly reported cause to remain in an abusive relationship. This might be due to the fact that historically, IPV was defined and studied in a heterosexual perspective, excluding any mention of same-gender relationships (Glass and Hassouneh, 2008; Little and Terrance, 2010). There are few existing examples of educational campaigns on LGB IPV, although the research proved how this kind of interventions is effective in encouraging battered people to report the abuse. Consequently, LGB partners involved in violence, and people close to them (services providers, family, friends), evaluated the battering as less dangerous or not harmful at all, and it usually took a longer time to recognize it as an abuse (Dixon and Peterman, 2003; Barrett, 2015).

## Sexual Minority Stress

Carvalho et al. (2011) argued that LGB people experience unique stressors related to the condition of being a part of a sexual minority. These stressors, that appear to be associated to IPV, reflected the experience of Sexual Minority Stress, a model developed by Meyer (2003) with regard to members of a stigmatized group who experienced unique and additional stressors that nobody outside the group could ever experience. This model included internalized stressors (internalized homophobia, disclosure, and stigma consciousness) and externalized stressors (actual experiences of violence, discrimination, and harassment) (Meyer, 2003). Research showed how internalized stressors were positively correlated to physical, sexual, and psychological IPV (Balsam and Szymanski, 2005; Bartholomew et al., 2006; Carvalho et al., 2011; Edwards and Sylaska, 2013); on the contrary, externalized stressors were not related to any form of IPV, particularly when they were considered with internalized minority stressors (Balsam and Szymanski, 2005; Bartholomew et al., 2006; Edwards and Sylaska, 2013).

Thus, studies mainly focused on internalized minority stressors, such as Internalized Homophobia, establishing that IPV perpetrators addressed their negative emotions, originally self-addressed as homosexuals, to their partners. People with internalized homophobia have been deprived by partners of positive emotions with regard to their sexual orientation and reinforced their sense of responsibility in provoking the abuse (Balsam and Szymanski, 2005; Carvalho et al., 2011). Carvalho et al. (2011) reported that internalized homophobia and IPV were related in lesbian couples and influenced by the quality of the relationship. Therefore, both couples’ variables and individual experiences were equally fundamental in understanding the homosexual IPV phenomenon (Balsam and Szymanski, 2005; Carvalho et al., 2011). Although the relationship between internalized homophobia and IPV was proven, data suggested that it was not strong (D’Lima et al., 2014). This result might be due to the fact that research participants showed low levels of internalized homophobia, because it is rare that LGB people with high levels of internalized homophobia would cooperate for any LGB study. A further cause could be that the sample comprised highly educated white people (Carvalho et al., 2011).

Two researchers reported that disclosure was positively related to the risk of physical and psychological IPV: Bartholomew et al. (2006) analyzed a sample comprising homosexual and bisexual men, while Carvalho et al. (2011) studied the phenomenon among lesbian women. Such findings may be due to the fact that being openly out implied a longer period of time of being victimized by the partner but also the opposite: a shorter time in LGB relationships could imply lower chances to be involved in an abusive one (Bartholomew et al., 2006; Carvalho et al., 2011). With regard to this last aspect, perpetrators could intimidate the victim by threatening to oust them in front of their family, employer, landlord, former partner, or current guardian of their children (Borne et al., 2007; Carvalho et al., 2011).

The Consciousness Stigma has been the last internalized minority stressor studied in relation to IPV. Carvalho et al. (2011) indicated that stigma consciousness increased the likelihood of IPV. IPV perpetrators and victims reported high stigma consciousness rates; thus, it can be assumed that IPV makes people more worried about stigma consciousness and that it is positively correlated to the tendency to ignore abuse in order to protect IPV victims from the homophobic legal system.

Such results match with high stigma consciousness rates in people who are expected to suffer discrimination and be forced to avoid discriminating situations (Pinel, 1999; Derlega et al., 2003). To what we know, literature offers several evidences regarding the connection between minority stressors and SSIPV. As mentioned earlier, internalized stressors and IPV were strongly correlated. Some studies (Balsam and Szymanski, 2005; Carvalho et al., 2011; Finneran and Stephenson, 2014) showed the existence of a relationship between experienced discrimination and a higher risk of IPV. On the other hand, studies on the relationship between experienced discrimination and risk of SSIPV victimization are contradictory: some indicated the existence of such a relationship (Carvalho et al., 2011; Andrews et al., 2014; Finneran and Stephenson, 2014), while some denied it (Barrett and St.Pierre, 2013; Andrews et al., 2014).

These findings suggest that the connection between discrimination about sexual orientation (based on other people emotions and beliefs) and IPV is not completely clear, but that a relation between victimization and individual feelings regarding one’s own sexual orientation (internalized homophobia and stigma consciousness) exists (Edwards et al., 2015). However, it should be noted that such considerations are the result of cross-sectional studies, thereby making it difficult to determine whether a factor developed prior to, during, or after the occurrence of IPV. This implies that it is important to be cautious in generalizing such findings; instead, further research must be conducted on predictors and associated factors (Edwards et al., 2015). Moreover, clinicians should be aware that minority stressors are one of the main obstacles for people who have experienced or are involved in IPV and seeking help, and what could assist them: it was proven that heterosexism exacerbates difficulties in reporting the abuse to the police and in accessing in services for LGB people (Carvalho et al., 2011). IPV victims can be reluctant in seeking legal assistance, fearing discrimination or adequate legal protection. Balsam (2001) observed that over 60% of lesbian women who were interviewed decided not to leave the abusive partner because of lack of resources, and a majority of the sample did not seek help in a women’s shelter. In line with Balsam (2001) and Buford et al. (2007) emphasize that services and shelters were often unprepared to support homosexual victims of IPV.

Overstreet and Quinn (2013) created the IPV Stigmatization Model to explain barriers to seeking help. The model described three aspects of the individual experience: “stigma internalization,” “anticipated stigma,” and “cultural stigma.” Stigma internalization referred to the impact of internalized negative beliefs regarding IPV, which can influence individuals’ help-seeking behaviors and psychological distress. Surviving IPV can cause guilt, shame, and self-blame, all of which are challenges in seeking help for decreased self-efficacy. Anticipated stigma, that also functions at the interpersonal level, was regarding concerns related to whether others will react with disapproval or rejection toward the survivor when they learn about the IPV, thereby affecting the decision to seek help. Lastly, cultural stigma referred to the notion that IPV victims provoked their own victimization.

## LGB IPV Assessment and Treatment

The first program for SSIPV was developed in United States and strictly connected or identical to the ones for heterosexual population (Dixon and Peterman, 2003; Ristock and Timbang, 2005). However, a specific risk was highlighted in considering IPV as a universal experience, since this assumption implicated that the treatment might be the same for each person (Ford et al., 2013). There were similar aspects between heterosexual and homosexual IPV relationships, therefore policies and services tailored for heterosexual may be helpful to design specific interventions for LGB population (Dixon and Peterman, 2003; Ristock and Timbang, 2005). Heterosexual model can be the starting point for treatments addressed to LGB people, who deserve interventions based on their own peculiar experiences and needs (Finneran et al., 2013).

Renzetti (1996) examined the outcomes of the application of an unspecific treatment that did not consider sexual orientation and gender. In that study, 566 North American services were involved, part of the *National Directory of Domestic Violence Programs*, mostly addressing IPV heterosexual victims. Almost 96% of the workers declared that they were indiscriminately welcoming and responsive to all kind of people seeking help, according to a non-discrimination policy. However, there was discord between the statements made by mental health providers and the victims’ reports. In fact, just one out of ten victims received particular care specifically directed to lesbian women, but the remainder claimed that operators did not make any effort to comply with their needs. Other researches (Giorgio, 2002; Helfrich and Simpson, 2006) conducted in the United States confirmed this condition: victims reported heterosexism, discrimination, stigma, ridicule, disbelief, additional abuse, and hostility from services. Cheung et al. (2009) conducted a global Internet-based study on Asian men accessing services as IPV victims. Authors reported that gay men were not perceived as domestic violence service consumers unless they were perpetrators (Cheung et al., 2009). On the other hand, lesbian women highlighted a heterosexist language adopted by emergency, primary care, and other service providers (Dixon and Peterman, 2003). It is considered that services are rarely available for LGB people, and when they are, it is often difficult to access them, particularly in rural areas (Jeffries and Kay, 2010; Ford et al., 2013). Thus, it appears clear how heterosexual IPV, widely studied, can be considered as a starting point to better investigate and address homosexual couple abuse, without overlooking LGB-specific factors (Finneran et al., 2013).

### LGB-Tailored Assessment

Because of the multiple barriers and the invisibility of the problem in the context of IPV services, the role of the victims’ health care providers is critical. While it was found that in the United States many emergency departments, shelters, agencies, and clinics had IPV advocacy programs, most of these programs historically failed in responding adequately to abuse in LGB groups (Brown and Groscup, 2009; Hines and Douglas, 2011; Armstrong et al., 2014). Goodman et al. (2015) contended that during initial steps, services providers should recognize the problem, offer empathic support, ensure safety, and help the victim gain distance from a dangerous situation. Healthcare workers should screen for IPV in LGB patients and understand how patterns of IPV are different for these patients (Banks and Fedewa, 2012; Armstrong et al., 2014): standard approaches to IPV screening may be ineffectual for LGB people (Cavacuiti and Chan, 2008). Ard and Makadon (2011) highlighted the need for a sensitive and accurate assessment, which they discussed through clinical, institutional, educational, and research suggestions. The authors indicated that providers must be alert to the possibility of IPV as a cause of distress and illness among their LGB patients. Thus, according to them, clinicians should first inquire about sexual orientation in a sensitive and open manner, rather than simply screening for IPV (Ard and Makadon, 2011). Further, clinicians must use an inclusive language, avoiding any type of homophobic attitude, beginning from the first contact with the client (Eliason and Schope, 2001; Finneran et al., 2013). Ard and Makadon (2011) also highlighted how assessing LGB IPV fulfilled an important educational role for their LGB clients, because of the invisible nature of the phenomenon. Merrill and Wolfe (2000) discussed “recognition failure” as the failure to recognize intimate violent behaviors and, therefore, to seek or offer help such because of widespread ignorance regarding SSIPV. Several authors support public and specialized education believing that it would reduce the incidence of this phenomenon, by promoting earlier help-seeking and strengthening informal and formal support systems for victims (McClennen, 2005; Borne et al., 2007).

Merrill and Wolfe (2000) recommended similar suggestions, considering that SSIPV assessment and treatment should include the following aspects:

(1) A specific training on assessing and responding to LGB IPV, because many providers did not accurately detect and compassionately respond as they did to heterosexual victims.
(2) Education regarding homophobia and heterosexism, which often led to the assumption that the violence was not as serious as in heterosexual cases, that it was more likely to be mutual, that the perpetrator was always a man and the victim was a woman, or that it was somehow easier for a victim of SSIPV to stop and leave the abusive relationship.
(3) The development of appropriate response protocols for law enforcement professionals. A case of inadequate attitude was offered by police officers, since they often did not recognize partners as members of a couple (particularly if partners defined themselves as roommates because they were scared) and did not know how to identify the abusers at an SSIPV crime scene, relying upon gender as the sole criteria. Consequently, in LGB IPV cases, officers frequently did not arrest anyone, arrested either party, or the wrong person.
(4) A combination of past and current history of IPV during the screening, in shelters and other agencies; this suggestion was made with the aim of a better understanding of violence forms and patterns of abuse.
(5) The development of individualized treatment plans that must include a safety plan (which comprised three steps according to the authors—the first step is to identify signs of escalation, the second one is to predict the next violent episode, and the third step is to plan how to respond self-protectively) and supportive psychotherapy, finalized to reinforce client’s strength and self-determination. The psycho-educational intervention could list and define abusive behaviors and perpetrator tactics, examining the psychological consequences of violence, describing the cycle of violence, and going beyond common prejudices regarding LGB IPV.
(6) An assessment of both partners’ HIV status, since it was proved that HIV status played an important role in remaining in abusive relationships. As an application of this suggestion, in 2013, Finneran et al. (2013) created a short form to screen SSIPV. The tool included additional domains of IPV not currently found in screening tools, such as monitoring behaviors, controlling behaviors, and HIV-related IPV.

### LGB-Tailored Treatments

Even if research testified serious lacks in existing services (Herrmann and Turell, 2008; Brown and Groscup, 2009; Hines and Douglas, 2011), Ristock and Timbang (2005) reported examples of innovative programs developed within LGB communities. They introduced different interventions compared to heterosexual IPV protocols, serving both survivors and perpetrators. For example, they offered batterer intervention programs as well as advocacy programs to help LGB people access the legal justice system (*The Los Angeles Gay and Lesbian Center*) (Ristock and Timbang, 2005). Further, two approaches focused on the specific needs of queer women in San Francisco were the one promoted by *The Queer Asian Women’s Shelter* (Chung and Lee, 1999) and the one from *Queer Asian and Pacific Islander Women* (Lee and Utarti, 2003): they attempted to better respond to IPV and address the complexities of being part of a small marginalized community such as the LGB one, teaching how to ask service providers for help. Similarly, the *Washington State Coalition Against Domestic Violence* developed a protocol for working with friends and family members of IPV victims. As the research highlights, most of the time, victims of violence asked friends and family for help before accessing services, thereby giving them a primary supporting role.

In certain cases, services were associated with community-based initiatives that involved holding workshops and forums to address healthy relationships (Cronin et al., 2017). Ristock and Timbang (2005) and highlighted how discussion on building healthy relationships appeared to be more welcomed from lesbian victims than support groups for survivors. This fact might be due to victims’ concerns regarding their privacy, which was protected during conversations on several topics connected to violence. Such discussion may explore other issues such as expectation in relationships, negotiating differences, power issues, and warning signs of abuse rather than identifying who experienced violence and respecting participants privacy. Another objective was also to shift from organizational interventions to a community-based prevention to support health relationships and provide information and prevention to lesbian communities (Fonseca et al., 2009; Ford et al., 2013). The variety of approaches presented attempt to better respond to local settings rather than standardizing programs (Hatzenbuehler et al., 2015).

Another attempt to provide adequate services to LGB people was made by The Violence Against Women Act (VAWA) in 2013 that allowed the creation of services in the United States that are specifically designed for LGB victims of IPV and a legislation with regard to their rights. The act explicitly included a non-discrimination clause that prohibited LGB individuals from being turned away from shelters or other programs funded by The Violence Against Women Act (Armstrong et al., 2014).

Further, several treatments and programs have been developed for individuals who experienced IPV. Some programs focused exclusively on treating the symptoms experienced by the victims, while others attempted to break the cycle of violence through interventions addressed for batterers. The types of interventions ranged from couple and group interventions to individual psychotherapy (Fountain and Skolnik, 2007; Herrmann and Turell, 2008; Dykstra et al., 2013; Armstrong et al., 2014; Quillin and Strickler, 2015).

### Couple and Group Interventions

Lesbian, gay, and bisexual partners often ask for treatment as a couple, and it is only after an initial assessment it becomes evident that the relationship is abusive. Frequently, with the aim of protecting victims, clinicians recommend separate services and refuse to provide couple therapy (Borne et al., 2007). In certain cases, this attitude was damaging and resulted in clients discontinuing treatment or seeking a different therapy (Istar, 1996). Merrill and Wolfe (2000) found that couple therapy was disadvantageous in IPV cases because it made it more difficult for victims to end the relationship and giving violence the label of “couple issues.” It also made it particularly difficult for the therapist to guarantee victims’ safety after therapy: occasionally, it created additional violence because of certain statements made by the therapist. Moreover, the authors indicated that couple therapy hindered an accurate assessment of the abuse because of victims’ fear of repercussions. In certain cases, it damaged partners because of therapist counter-transference, who believed it was right to punish the violent person in the couple in order to protect the victim instead of sticking to therapy (Merrill and Wolfe, 2000).

Dykstra et al. (2013), in their review on IPV treatment effectiveness, found that couple therapy can be an effective treatment and it is occasionally necessary, particularly during the initial phases, to adequately assess the dynamics of the relationship. Moreover, an accurate assessment of the violence and the associated risks should be required in considering couple violence as a treatment option; this would enable the provision of the most suitable assistance for the couple in terms of defining or redefining problems, which can be treated through individual treatment plans (Borne et al., 2007). Couples therapy can provide a safe space where relationships can be discussed and negotiated (Gilbert et al., 2017). On the other hand, couples therapy can be self-defeating if one or both of the partners presents issues that would best be previously acknowledged through individual counseling (Borne et al., 2007).

The effectiveness of couple therapy increased when combined with either individual or group therapy (Ristock and Timbang, 2005; Gilbert et al., 2017). Coleman (2003) highlighted that the optimal treatment for perpetrators is group therapy combined with long-term psychoanalytic psychotherapy or psychoanalysis.

Dykstra et al. (2013) evidenced that group therapy can be extremely useful in treating IPV and create room for improving emotional and social functioning. Group therapy made it possible to experience support and confrontation in a safe space, thereby avoiding isolation—a common consequence of victimization. The peer group assisted individuals with reliability by challenging unhealthy conduct and encouraging healthy behaviors. On the other hand, perpetrators too had the opportunity to learn new cognitive and behavioral strategies for managing their abusive impulses and express their emotions in a functional and structured manner (Buttell and Cannon, 2015). Occasionally, in case patients refuse to participate in group therapy, group therapy activities can be adapted to individual cases. Coleman (2003) listed some specific techniques: time-outs, control logs, and the Iceberg Exercise (that helped patients to identify emotions underlying their anger).

### Victims’ Treatments

A few studies on treatment for LGB IPV victims were conducted in the United States (Browning et al., 1991; McClennen et al., 2002; Dixon and Peterman, 2003; Buford et al., 2007; Fountain and Skolnik, 2007; Ard and Makadon, 2011; Franklin and Jin, 2016). Studies showed that individual mental health counseling can result in good outcomes for SSIPV victims. Couple counseling with victim and abuser was found to be less effective because victims may fear repercussions from the information given during the session (such as details of the victimization) (Buford et al., 2007; Winstead et al., 2017). In spite of these findings, research has indicated that psychology graduate students and clinicians have the inclination to suggest couples counseling instead of individual counseling for LGB IPV victims more often than for different-gender victims (Wise and Bowman, 1997; Poorman et al., 2003).

Two types of counseling proposed as ideal for SSIPV victims were the person-centered approach and Gestalt therapy. These approaches allowed victims to gradually feel more trustful toward therapists and thus become aware of their status, the suffered abuse, and the associated consequences to it (Dietz, 2002). Moreover, it encourages therapists to enable victims to direct the session, thereby learning, in this manner, how to effectively direct their lives. Dixon and Peterman (2003) found that because of the strong motivation to accept help, victims’ awareness about the abuse was believed to be longer-lasting. This fact granted victims to gain and adopt useful resources to bring an end to the abusive condition and obtain independence from the partner.

### Interventions Addressed to the Abusers

In the United States, it is not unusual for abusers to participate in psycho-educative programs finalized to reduce the risk of committing violence on partners in the future. These programs are called “batterer intervention programs” and are based on the following two models (Price and Rosenbaum, 2009; Buttell and Cannon, 2015):

• Cognitive Behavioral Therapy (CBT), which aims to stop violent inclinations and build useful resources directed to solve couple issues.
• The Duluth Model, finalized to disassemble and eliminate patriarchal beliefs in abusive men who were consequently encouraged to feel that they are right to control women.

Dykstra et al. (2013) highlight that the Duluth model, alone or combined with CBT techniques, was the most frequently used program in the treatment of abusers. Both approaches do not consider the peculiarities of LGB couples and the role played by factors such as homophobia (Buttell and Cannon, 2015).

Moreover, the Duluth model, based on the patriarchal ideology, was originally designed just for heterosexual couples; however, it was subsequently applied to LGB perpetrators (although in the United States the groups, during the treatment, were often separated according to sexual orientation, even if the programs were mostly the same for both groups) (Price and Rosenbaum, 2009; Buttell and Cannon, 2015). This feminist psycho-educational approach is focused on re-education toward the development of more adaptive attitudes, improving communication proficiency, and ultimately eliminating violent behaviors (Buttell and Cannon, 2015). To the best of our knowledge, there are no studies to test the impact of such treatment on the LGB population (Stith et al., 2012) and the few researches on heterosexual population show limited positive effects (Babcock et al., 2004; Stith et al., 2012). Buttell and Cannon (2015) stated that scholars applying a post-structuralist feminist framework to IPV argued that a one-size-fits-all treatment model for IPV perpetrators (e.g., the Duluth model) should be replaced by culturally relevant and specific treatment options for LGB perpetrators. In their opinion, treatment interventions should address issues of sexism, homophobia, racism, and classism in order to address the ways society materially disadvantages some while privileging others (Buttell and Cannon, 2015).

Cannon et al. (2016) analyzed 3,246 questionnaire sent to directors of domestic violence perpetrator programs in the North American Domestic Violence Intervention Program Survey, in the United States and Canada. The results highlight that the most common approach to LGB batterers was a one-to-one approach instead of a group therapy, due to the difficulties for LGB people to express openly express themselves in heterosexual groups, two programs were projected for the LGB population.

### Cross-National/Cross-Cultural Differences

Many interventions were developed in the North American context (Istar, 1996; Merrill and Wolfe, 2000; Dixon and Peterman, 2003; Lee and Utarti, 2003; Ristock and Timbang, 2005; Borne et al., 2007; Fountain and Skolnik, 2007; Herrmann and Turell, 2008; Price and Rosenbaum, 2009; Hines and Douglas, 2011; Dykstra et al., 2013; Armstrong et al., 2014; Buttell and Cannon, 2015; Quillin and Strickler, 2015), while a few existed in Canada (Senn and St.Pierre, 2010; Cannon et al., 2016; Barata et al., 2017) and Australia (Leonard et al., 2008; Jeffries and Kay, 2010). Some interventions were addressed to a specific ethnic group, such as Asians (Chung and Lee, 1999; Lee and Utarti, 2003; Cheung et al., 2009), or black people (Helfrich and Simpson, 2014). Moreover, IPV services where more accessible in urban centers where the LGB community was well developed and rooted than in rural areas (Jeffries and Kay, 2010; Ford et al., 2013). To the best of our knowledge, specific researches have addressed to IPV assessment/treatment for the LGB population in other countries.

## Access to Services Offering Help and Support

Because of the impact of homophobia, homosexual and bisexual people may have a significantly more difficult time finding and receiving appropriate help than heterosexual ones, particularly when other variables such as income, ethnicity, and immigration status were held constant (Ard and Makadon, 2011; Barata et al., 2017).

Lesbian, gay, and bisexual victims of IPV access treatments through a wide range of help-giving resources, which can be distinguished into informal (family, friends, acquaintances) and formal resources (support groups, LGB community agencies, hotlines and shelters for IPV victims, medical health-care providers, and the criminal justice system). LGB victims of IPV were prone to seek help from informal resources (particularly friends) (Scherzer, 1998; Merrill and Wolfe, 2000; Turell, 2000), although there was a rather high percentage of people who turned to health care providers and family (Scherzer, 1998; Merrill and Wolfe, 2000; Turell, 2000); on the contrary, organizations specifically designed with the purpose of addressing IPV seemed to have the lowest utilization rates (Lanzerotti, 2006). In terms of the gender of the victim, it emerged that lesbian women had the tendency to seek help from all types of resources equally, while gay men were more prone to turn to the police to report victimizations (Cornell-Swanson and Turell, 2006; Senn and St.Pierre, 2010).

These results confirmed the need for specific interventions for LGB people, particularly considering that the health system offered low quality support, beginning from the fact that health professionals who assessed heterosexual female patients for IPV typically did not similarly screen lesbian or bisexual female patients or male patients of any sexual orientation in the same manner (Jeffries and Kay, 2010; O’Neal and Parry, 2015; Barata et al., 2017). McClennen et al. (2002) identified that a 7–33% of the victims evaluated the health system support as valid. Several studies highlighted that many interventions were perceived as unsatisfying because of homophobic (Tigert, 2001; Helfrich and Simpson, 2006, 2014) or superficial attitudes, denying the seriousness of the violence—“women are not as violent to one another” and “men can protect themselves” (Chung et al., 2008; Fonseca et al., 2010). These findings are consistent with Seelau and Seelau (2005) that considers perpetrators as more aggressive if the victim was a woman instead of a man. Male perpetrators were judged more blame-worthy than female perpetrators. Overall, male–female IPV was considered more dangerous than female–male, male–male, or female–female abuse. Significantly, the gender of the survivor, not sexual identity, was the most prominent factor in predicting witness response. In accordance with this, Arnocky and Vaillancourt (2014) work suggested that men, regardless of sexual identity, were less likely to recognize that they were being abused than women. To date, trainings on LGB IPV received by operators appear to be lacking, while the operators often believe to have an appropriate competence regarding heterosexual IPV (Senn and St.Pierre, 2010; Hancock et al., 2014).

## Conclusion

The literature on LGB IPV is recent and limited compared to the one on heterosexual IPV. However, a growing body of empirical research does exist, thereby offering important observations and considerations regarding LGB IPV. Previous studies primarily examined the prevalence of IPV in the homosexual and bisexual population (Turell, 2000; Messinger, 2011; Barrett and St.Pierre, 2013; Breiding et al., 2013), LGB specific features in IPV (Merrill and Wolfe, 2000; Balsam and Szymanski, 2005; Bartholomew et al., 2006; Carvalho et al., 2011; Edwards and Sylaska, 2013; Gill et al., 2013) and barriers to treatment (McClennen et al., 2002; Ard and Makadon, 2011). There are only a few publications on treatments and interventions for LGB IPV (Browning et al., 1991; McClennen et al., 2002; Dixon and Peterman, 2003; Ristock and Timbang, 2005; Buford et al., 2007; Fountain and Skolnik, 2007; Herrmann and Turell, 2008; Ard and Makadon, 2011; Quillin and Strickler, 2015). They can be classified into counseling interventions, particularly for victims (Dietz, 2002; Dixon and Peterman, 2003; Poorman et al., 2003; Buford et al., 2007; Franklin and Jin, 2016), and therapy: couple (Istar, 1996; Borne et al., 2007; Dykstra et al., 2013; Buttell and Cannon, 2015), group (Coleman, 2003; Ristock and Timbang, 2005; Buttell and Cannon, 2015), and for perpetrators (Babcock et al., 2004; Dykstra et al., 2013; Buttell and Cannon, 2015).

Despite the myth that IPV is only an issue in heterosexual relationships, its occurrence among LGB couples was demonstrated to be comparable to or higher than heterosexual cases (Messinger, 2011; Kelley et al., 2012; Barrett and St.Pierre, 2013; Breiding et al., 2013). While similarities between heterosexual and LGB IPV (such as general patterns, types, outcomes, cycle of violence and use of substances) were found (McLaughlin and Rozee, 2001; Buford et al., 2007; Cain et al., 2008; Hequembourg et al., 2008), unique features and dynamics were present in LGB IPV, which were implicated in identifying and treating IPV among the community (Merrill and Wolfe, 2000; Carvalho et al., 2011; Bowen and Nowinsky, 2012; Gill et al., 2013).

Even though literature on LGB IPV is lacking in general, there is a need for research specifically on treatment (Dupont and Sokoloff, 2005). Results suggested that several obstacles prevent LGB people from getting help in case of IPV (Alhusen et al., 2010; O’Neal and Parry, 2015), heterosexism above all. IPV victims can be reluctant in seeking assistance, fearing discrimination (Giorgio, 2002; Helfrich and Simpson, 2006; Carvalho et al., 2011). Rarely a solution was offered to help LGB people in accessing treatment for IPV, and authors recommended modifications to standard treatments or programs (Calton et al., 2015; O’Neal and Parry, 2015). Studies showed that services and shelters were often unprepared to support IPV homosexual and bisexual victims (Buford et al., 2007; Cannon et al., 2016; Barata et al., 2017). In the United States, many emergency departments, shelters, agencies, and clinics had IPV advocacy programs; most of these programs historically failed in responding adequately to abuse in LGB groups (Brown and Groscup, 2009; Ford et al., 2013; Armstrong et al., 2014). The majority of the researches takes into consideration only North American services and programs existing in urban centers, while rural areas or other countries were not investigated (Jeffries and Kay, 2010; Ford et al., 2013). Comparing the few programs specializing in SSIPV treatment to traditional protocol, they were modified in assessing processes for sexual identity, in helping SSIPV victims in accessing the legal justice system, and in avoiding stigmatization (Merrill and Wolfe, 2000; Ristock and Timbang, 2005; Armstrong et al., 2014; Cannon et al., 2016). However, studies did include recommendations in order to focus on LGB-specific treatment. While many researchers recommended modified versions of IPV treatment, no one empirically studied whether LGB people benefit more from modified versions of treatment than standard treatments (Stith et al., 2012).

It is crucial to address an additional issue related to the cultural and social context: the fact that we found studies on treatment only in the North-American context indicates a lack of research in this field in other countries; however, it is possible that some studies were not present in international databases. The reviewed literature suggested the need of a psychological treatment designed on specific LGB necessities and finalized to guarantee new useful resources and develop self-determination (Merrill and Wolfe, 2000; Calton et al., 2015; O’Neal and Parry, 2015). Intervention for LGB IPV victims and perpetrators should be part of an integrate and complete treatment plan that can involve couples or individual treatment but, in any case, that should be adapted to each specific situation. In line with such considerations, adequate training for mental health providers and standard guidelines for assessment and treatment may lead to more positive outcomes. Improvements should concern victims’ well-being and satisfaction and treatment features, such as the durability of the treatment effects; moreover, a new approach may define an easier accessibility to services (Alhusen et al., 2010; Ard and Makadon, 2011; Banks and Fedewa, 2012). Since IPV appears to be an issue as common and serious in same-gender relationships as in heterosexual ones, policies and practices should update to guarantee the same degree of protection (Brown, 2008).

Because of the lack of program specialized in addressing SSIPV it would be important that the emerging IPV programs should provide outreach and educational services by cooperating with the community and offering several services, beginning from direct and physical resources such as shelters, food and clothing, transportation, financial and legal assistance, 24-h hotlines and individual and group therapy. Although traditional battered women’s shelters can be recognized as a model for LGB agencies, some changes should be made: for example, a more inclusive language and a focus on experiences of individuals rather than gender, which can make LGB people more comfortable in disclosing abuse. IPV is still a partially unknown issue in the LGB community, which may minimize warning signs and this is why the LGB community needs to be specifically targeted for education regarding IPV and recognize its signs (Coleman, 2003; Dixon and Peterman, 2003; Dutton et al., 2009; Ard and Makadon, 2011; Bowen and Nowinsky, 2012; Calton et al., 2015; O’Neal and Parry, 2015; Cannon et al., 2016).

## Author Contributions

GG and LR took overall responsibility for the creation of the frame used in this review and the selection of the papers. GG, AC, and EG searched for the articles discussed in the review. LR and PB supervised the entire work. All the authors were involved in the discussion, writing and revision of the manuscript, and approved the final version of the manuscript to be published.

## Conflict of Interest Statement

The authors declare that the research was conducted in the absence of any commercial or financial relationships that could be construed as a potential conflict of interest.
