# Corrigendum: When Intimate Partner Violence Meets Same Sex Couples: A Review of Same Sex Intimate Partner Violence

**DOI:** 10.3389/fpsyg.2019.01706

**Published:** 2019-07-19

**Authors:** Luca Rollè, Giulia Giardina, Angela M. Caldarera, Eva Gerino, Piera Brustia

**Affiliations:** Department of Psychology, University of Torino, Turin, Italy

**Keywords:** same sex intimate partner violence, same-sex couple, LGB, domestic violence, IPV, treatment

In the original article, there was an error. The life-time prevalence of IPV in heterosexual women was incorrectly provided.

A correction has been made to the ***Introduction***, paragraph three:

“Life-time prevalence of IPV in LGB couples appeared to be similar to or higher than in heterosexual ones: 61.1% of bisexual women, 43.8% of lesbian women, 37.3% of bisexual men, and 26.0% of homosexual men experienced IPV during their life, while 35.0% of heterosexual women and 29.0% of heterosexual men experienced IPV. When episodes of severe violence were considered, prevalence was similar or higher for LGB adults (bisexual women: 49.3%; lesbian women: 29.4%; homosexual men: 16.4%) compared to heterosexual adults (heterosexual women: 23.6%; heterosexual men: 13.9%) (Breiding et al., 2013).”

The authors apologize for this error and state that this does not change the scientific conclusions of the article in any way. The original article has been updated.
