# Corrigendum: When intimate partner violence meets same sex couples: a review of same sex intimate partner violence

**DOI:** 10.3389/fpsyg.2024.1449682

**Published:** 2024-07-09

**Authors:** Luca Rollè, Giulia Giardina, Angela M. Caldarera, Eva Gerino, Piera Brustia

**Affiliations:** Department of Psychology, University of Torino, Turin, Italy

**Keywords:** same sex intimate partner violence, same-sex couple, LGB, domestic violence, IPV, treatment

In the published article, there was an error in the Introduction. The percentages reported from Breiding et al. (2013) were incorrect. The sentence previously stated, “over 50% of gay men and almost 75% of lesbian women reported that they were victims of psychological IPV (Breiding et al., 2013).”

A correction has been made to **Introduction**, Paragraph 2. The corrected sentence is shown below.

“In addition, around 60% of gay men and 63% of lesbian women reported that they experienced psychological IPV (Breiding et al., 2013).”

The authors apologize for this error and state that this does not change the scientific conclusions of the article in any way. The original article has been updated.

